# ERCC1 mRNA levels can predict the response to cisplatin-based concurrent chemoradiotherapy of locally advanced cervical squamous cell carcinoma

**DOI:** 10.1186/1748-717X-7-221

**Published:** 2012-12-23

**Authors:** Zhou-lan Bai, Yan-yang Wang, Hong Zhe, Jian-li He, Ping Hai

**Affiliations:** 1 Graduate School, Ningxia Medical University, No.1160 Shengli Str, Yinchuan, Ningxia, 750004, China; 2 Department of Radiation Oncology, General Hospital of Ningxia Medical University, No.804 Shengli Str, Yinchuan, Ningxia, 750004, China

**Keywords:** Excision repair cross-complementation group 1 (ERCC1), Cervical squamous cell carcinoma, Chemoradiotherapy, Response prediction

## Abstract

**Background:**

The purpose of this study was to investigate whether the excision repair cross-complementation group 1 (ERCC1) mRNA expression could predict treatment response of patients with locally advanced cervical squamous cell carcinoma (LACSCC) who underwent cisplatin-based concurrent chemoradiotherapy (CCCRT).

**Methods:**

A total of sixty LACSCC patients, treated with radical CCCRT from a single institution were evaluated. ERCC1 mRNA expression was determined by quantitative real-time RT-PCR in pre-treatment tumor tissues. The association of ERCC1 status with clinicopathological characteristics (age, histological grade, tumor size, parametrial invasion, lymph node metastasis and FIGO stage) and treatment response were analyzed.

**Results:**

No significant association between ERCC1 mRNA expression and clinicopathological characteristics were observed. Patients with low ERCC1 mRNA level had a significantly higher rate of complete response (86.21%) than patients with high level of ERCC1 expression (19.36%; p < 0.001). In the logistic regression analysis, low ERCC1 mRNA level retained an independent role in predicting complete response to CCCRT (*P* < 0.001). An ERCC1 expression level of 0.0901 was determined as an optimal cutoff value to identify complete response patients to CCCRT treatment. The sensitivity for detection of a complete response was 81.48% with a specificity of 96.97% (area under the curve, 0.893; 95% confidence interval, 0.804–0.983).

**Conclusions:**

This is the first analysis of the association between ERCC1 mRNA levels and treatment response in patients with LACSCC. Low ERCC1 mRNA level appears to be a highly specific predictor of response to CCCRT in LACSCC.

## Background

The current standard treatment of locally advanced cervical cancer is cisplatin-based concurrent chemoradiotherapy (CCCRT), followed by brachytherapy [1]. However, individual patients may show quite different patterns of response against CCCRT; some can be cured, but others cannot, and the latter may therefore suffer severe side effects [2]. Identification of biomarkers which predict response to CCCRT will allow patients to avoid toxicity associated with the ineffective therapy.

One of the most promising biomarkers for response prediction is the excision repair cross-complementation group 1 (ERCC1). ERCC1, which is involved in nucleotide excision repair and associated with resistance to cisplatin-based chemotherapy or chemoradiotherapy in various types of cancer [3-10]. Earlier in vitro studies have linked cisplatin resistance to the expression of ERCC1 mRNA in cell lines of cervical cancers [11]. In a murine xenograft model, upregulated ERCC1 expression is associated with radioresistance in tumors derived from cervical carcinoma cells [12]. However, the association of ERCC1 and treatment response in clinical setting, especially for cervical squamous cell carcinoma, has not be well documented. The purpose of present study was to evaluate whether the ERCC1 mRNA expression levels could predict the treatment response of patients with locally advanced cervical squamous cell carcinoma (LACSCC) who underwent CCCRT.

## Patients and methods

### Patients and samples

Cervical squamous cell carcinoma tissues and corresponding non-tumorous tissues were obtained with informed consent from sixty consecutive LACSCC patients who underwent biopsy before CCCRT at Ningxia Medical University General Hospital, between June 2009 and June 2010, and frozen in liquid nitrogen until further analysis. Patients had a median age of 53 years (range 36 to 80 years) and no patient received previous radiotherapy or chemotherapy. Histologically, all primary tumors were squamous cell carcinoma. Staging was performed according to International Federation of Gynecology and Obstetrics (FIGO) staging system classification. Clinicopathological characteristics of these patients are listed in Table 1. This study was approved by the ethics committee of our hospital.

**Table 1:** Clinicopathological characteristics of the patients with LACSCC according to the ERCC1 mRNA levels

**Characteristic**	**ERCC1 mRNA**		***P***
	**High level**	**Low level**	
Age, y			
≤50	14	13	
>50	15	18	0.622
Histologic grade			
Well and moderately differentiated	12	19	
Poorly differentiated	17	12	0.123
Tumor size, cm			
≤4	8	12	
>4	21	19	0.361
Parametrial invasion			
No	14	15	
Unilateral	12	11	
Bilateral	3	5	0.774
Clinical lymph node involvement			
N0	18	26	
N1	11	5	0.056
FIGO stage			
II	14	22	
III	15	9	0.073
Hemoglobin levels at diagnosis, g/dL			
≤11.3	8	5	
>11.3	21	26	0.282
Platelets at diagnosis, ×10^9^/L			
≤320	26	24	
>320	3	7	0.204

*Abbreviations: LACSCC* locally advanced cervical squamous cell carcinomas, *ERCC1* excision repair cross-complementation group 1, *FIGO* International Federation of Gynecology and Obstetrics.

### Treatment and response

The pretreatment evaluation included a review of the patient’s history, physical examination, performance status, biopsy and gynecologic examination under general anesthesia, chest X-ray, complete blood count, blood chemistry, and abdominal-pelvic magnetic resonance imaging. Cystoscopy and sigmoidoscopy were performed when indicated.

Radiotherapy included external beam radiotherapy up to 50 Gy and low-dose rate brachytherapy, six applications of 6 Gy. Chemotherapy consisted of weekly intravenous cisplatin administration (40 mg/m^2^) for 5 cycles concomitant with external pelvic radiation. Treatment response was clinically assessed according to the RECIST criteria [13]. We categorized patients into two response groups: the sensitive group and the resistant group. The sensitive group included those patients who achieved a complete response (CR as indicated in the RECIST criteria) and remained in remission throughout the follow-up period. The resistant group included patients who had persistent disease (PR, SD and PD as indicated in the RECIST criteria) after treatment or developed a relapse after remission.

### RNA Extraction, reverse transcription and quantitative real time RT-PCR (QRT-PCR) assays

RNA was isolated using the Trizol reagent (Invitrogen, Carlsbad, CA, USA) following the manufacturer’s protocol from the cancerous and corresponding non-tumorous tissues. Concentration of total RNA was estimated by a SmarSpec Plus spectrophotometer (BIO-RAD, Hercules, CA,USA) and stored at −80°C.

After treatment with DNAfree (Ambion, Austin, TX, USA) to remove chromosomal DNA, complementary DNA (cDNA) was synthesized using SuperScript III Reverse Transcriptase (Invitrogen, Carlsbad, CA, USA) and stored at −20°C until use.

The mRNA expression levels of ERCC1 and beta-actin were measured by quantitative RT-PCR using IQ 5 Multicolor Real-Time PCR Detection Systerm (BIO-RAD, Hercules, CA, USA). The cycling conditions were as follows: 10 min of an initial denaturation step at 95°C, followed by 40 cycles of 30 sec at 95°C, 30 sec at 58°C and 30 sec at 72°C. The following primers were used: ERCC1, forward: CCTCAGACCTACGCCGAATA; reverse: GCTCACAATGATGCTGTTGG [14]; and beta-actin, forward: TGACGTGGACATCCGCAAAG; Reverse: CTGGAAGGTGGACAGCGAGG.PCR products were scanned, and quantification was performed by the Quantity One program (Bio-Rad, Hercules, CA, USA). The expression of beta-actin was used as an internal control. The ERCC1 expression level was normalized to the beta-actin mRNA level using the 2^-△△Ct^ method [15].

### Statistical analysis

The median relative ERCC1 mRNA expression level standardized for beta-actin was selected as cut-off value of high and low level ERCC1 expression. Associations between dichotomized ERCC1 mRNA levels and clinicopathological characteristics were assessed for statistical significance using a chi-square test. Logistic regression models were used to identify independent predictive factors for treatment response. Cut-off values for discrimination of ERCC1 mRNA levels and treatment response were derived from receiver operating curve data (ROC; area under the curve and the 95% confidence interval). All reported *P* values are two-sided, and *P* less than 0.05 was considered statistically significant. SPSS 13.0 (SPSS Inc., Chicago, IL) was used for the statistical analysis.

## Results

ERCC1 mRNA expression levels were successfully measured in sixty patients. The median measured value of ERCC1 mRNA was 23.06 (range, 18.78–25.46). The median relative ERCC1 mRNA expression level standardized for beta-actin was 0.0347 (range, 0.0028–5.4264). Thirty-one (51.67%) patients were classified as having high level. No significant differences were found in the clinicopathological characteristics between the patients with high ERCC1 mRNA level and those with low level (Table 1).

**Figure 1:**
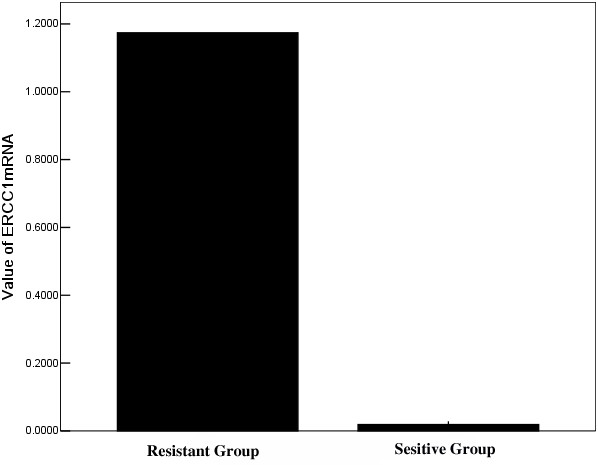
The mean value of ERCC1 mRNA expression between sensitive and resistant groups.

All patients received the external beam radiotherapy and brachytherapy as indicated in the protocol. For myelosurppression and infectious complications, only 34 (56.67%) patients received 5 cycles cisplatin-based concurrent chemotherapy. The treatment response was evaluated by CT imaging after treatment completion. Of the 60 patients who entered the study, 33 (55%) patients had complete response. Patients with low ERCC1 mRNA level had a significantly higher complete response rate (86.21%) than patients with high expression levels (19.36%; p < 0.001) (Figure 1). In the logistic regression analysis, only low ERCC1 mRNA level retained an independent role in predicting complete response to CCCRT (Table 2).

**Table 2:** Results of logistic regression analysis for clinical factors and response to CCCRT

	**B**	**SE**	**Wald**	**P value**	**OR**	**95% CI for OR**
Age	−0.104	0.064	2.599	0.107	0.902	0.795-1.023
Histologic grade	−0.450	1.090	0.171	0.680	0.638	0.075-5.399
Tumor size	2.908	1.567	3.445	0.063	18.315	0.850-394.766
Parametrial invasion	2.773	1.878	2.180	0.140	16.011	0.403-635.705
Lymph node status	−0.274	1.060	0.067	0.796	0.760	0.095-6.074
FIGO stage	2.471	1.797	1.891	0.169	11.836	0.349-400.843
Hemoglobin levels at diagnosis	−0.326	1.252	0.068	0.795	0.722	0.062-8.398
Platelets at diagnosis	−0.166	1.309	0.016	0.899	0.847	0.065-11.013
ERCC1 mRNA status	5.003	1.408	12.624	0.000	148.869	9.424-2351.736
Chemotherapy cycles	−0.932	1.073	0.755	0.385	0.394	0.048-3.225

*Abbreviations: CCCRT* cisplatin-based concurrent chemoradiotherapy, *B* regression coefficient, *SE* standard error, *Wald* Wald value, *OR* odds ratio, *CI* confidence interval, *FIGO* International Federation of Gynecology and Obstetrics, *ERCC1* excision repair cross-complementation group 1.

**Figure 2:**
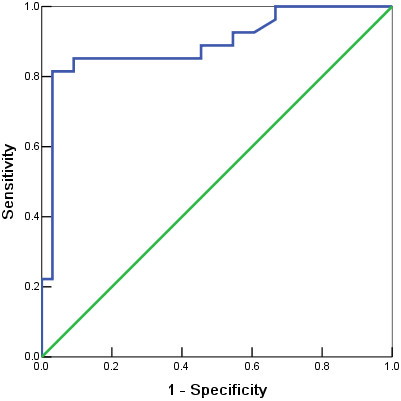
ROC curve depicting sensitivity and specificity of ERCC1 mRNA levels in predicting complete response of CCCRT.

ROC analysis was applied to determine an ERCC1 mRNA value that best segregates patients into complete response or non-complete response. An ERCC1 expression level of 0.0901 was determined as an optimal cutoff value to identify complete response to CCCRT treatment. The sensitivity for detection of a complete response was 81.48% with a specificity of 96.97% (area under the curve, 0.893; 95% confidence interval, 0.804–0.983) (Figure 2).

## Discussions

The addition of concurrent cisplatin-based chemotherapy to standard radiotherapy could reduce the risk of recurrence and disease-related death rates from locally advanced cervical cancer by as much as 50% [16,17]. However, those without a response will have toxicity helplessly. It would be useful to predict the response and to search for novel approaches for non-responders [18].

ERCC1 plays an important role in recognizing and removing cisplatin–induced DNA adducts and repairs interstrand cross-links in DNA and recombination processes [19,20]. Several preclinical and clinical studies have investigated the expression of ERCC1 mRNA and protein in multiple cancer types and have demonstrated a correlation between the ERCC1 expression levels and the treatment response to platinum-based therapy and/or survival outcome [3-10]. These results suggest that ERCC1 expression may assist in selecting patients most likely to benefit from platinum agent-based chemotherapy or chemoradiotherapy.

There are three other studies evaluated the relationship of ERCC1 status and the treatment response or survival of the patients with cervical carcinoma who received radiotherapy or chemoradiotherapy. Liang et al. assessed the ERCC1 expression of 50 patients with cervical squamous cell carcinomas who received cisplatin-based concurrent chemoradiotherapy by immunohistochemistry (IHC) method. They found ERCC1-negative patients had a significantly higher complete response rate than ERCC1-positive patients (*P* = 0.015). The 5-year overall survival (OS) rates for the ERCC1-positive and ERCC1-negative groups were 50.0% and 85.3%, respectively. For OS, lack of ERCC1 expression was an independent prognostic factor [21]. Doll et al. evaluated the association of ERCC1 expression, using both mRNA and protein expression analysis, with clinical outcome in cervical cancer patients treated with radical radiotherapy. ERCC1 mRNA level was determined by real-time PCR, and ERCC1 protein expression (FL297, 8F1) was measured using quantitative IHC. ERCC1 protein expression levels using both FL297 and 8F1 antibodies were determined for 112 patients; mRNA analysis was performed in 32 patients. In the 112 patients, 99 patients were squamous cell carcinomas, and 33 patients were adenocarcinomas. Low ERCC1 mRNA expression status was associated with worse OS (p = 0.046). ERCC1 protein expression using the FL297 antibody, but not the 8F1 antibody, was significantly associated with both OS (p = 0.002) [22]. Hasegawa et al. analyzed the ERCC1 expression of 36 patients with cervical adenocarcinoma by IHC method. Among the 25 patients who received cisplatin-based chemotherapy or chemoradiotherapy with cisplatin, those with high ERCC1 expression experienced significantly worse disease-free survival than those with low ERCC1 expression (*P* = 0.002). Moreover, univariate and multivariate analyses revealed that high ERCC1 expression was an independent prognostic factor in patients receiving cisplatin-based chemotherapy or chemoradiotherapy with cisplatin [23]. Our study is the first analysis of the association between ERCC1 mRNA expression and treatment response in patients with cervical squamous cell carcinomas who received radical cisplatin-based concurrent chemoradiotherapy. Sixty patients were evaluated in this study, which is the largest number among these studies. We firstly found that cervical squamous cell carcinomas patients with low ERCC1 mRNA level had a significantly higher rate of complete response than patients with high level of ERCC1 expression (*P* < 0.001). In the logistic regression analysis, low ERCC1 mRNA level retained an independent role in predicting complete response to CCCRT (*P* < 0.001).

Although the results of our study are similar to Liang’s and Hasegawa’s study, which are opposite to Doll’s study. The first reason of these differences is the histology type of enrolled patients was different. Squamous cell carcinoma comprises around 85% to 90% of uterine cervical cancer [24,25]. It has different biology behavior and treatment response when compared with adenocacinoma [2,26]. In Doll’s study, there are 11% patients with cervical adenocarcinoma. The second reason is that the treatment method was different between two studies. In Doll’s study, they used radiation alone. In fact, ERCC1 expression and the response of chemotherapy also have interaction [10].

There are some shortages in this study. Firstly, we did not perform IHC. High ERCC1 mRNA only signifies ERCC1 DNA transcription and does not necessarily reflect the production of functional ERCC1 protein, which could be assessed by IHC. We will evaluate the ERCC1 protein expression and the treatment response of LACSCC in further study. Secondly, the number of patients was small. We need do further research to validate the results in large number patients. Lastly, due to the short follow-up time, the association of ERCC1 mRNA levels and survival was not performed in present study at this point.

## Conclusions

This is the first study to evaluate the relationship of ERCC1 mRNA levels and complete response of LACSCC who underwent CCCRT. We have shown that ERCC1 expression patterns in pretreatment cancerous specimens can be effectively used to predict response to CCCRT. These results suggest that ERCC1 expression may assist in selecting patients most likely to benefit from CCCRT. Further investigation is required to determine whether these assays are sufficiently reliable to use routinely as a basis to select specific patient treatments.

## Competing interests

The authors declare that they have no competing interests.

## Authors’ contributions

ZL Bai and YY Wang participated in acquiring clinical and laboratory data, data analysis and interpretation, acquiring clinical samples, follow-up clinical information and final writing of the manuscript. H Zhe, JL He and P Hai participated in acquiring clinical and laboratory data, data analysis and data interpretation and drafted the manuscript. All authors read and approved the final manuscript.
